# Death-Associated Protein Kinase 1 Phosphorylation in Neuronal Cell Death and Neurodegenerative Disease

**DOI:** 10.3390/ijms20133131

**Published:** 2019-06-26

**Authors:** Nami Kim, Dongmei Chen, Xiao Zhen Zhou, Tae Ho Lee

**Affiliations:** 1 Division of Gerontology, Department of Medicine, Beth Israel Deaconess Medical Center, Harvard Medical School, Boston, MA 02215, USA; 2 Fujian Key Laboratory for Translational Research in Cancer and Neurodegenerative Diseases, Institute for Translational Medicine, School of Basic Medical Sciences, Fujian Medical University, Fuzhou 350122, China; 3 Division of Translational Therapeutics, Department of Medicine and Cancer Research Institute, Beth Israel Deaconess Medical Center, Harvard Medical School, Boston, MA 02215, USA

**Keywords:** death-associated protein kinase 1 (DAPK1), Alzheimer’s disease (AD), ischemic stroke, neuronal cell death, phosphorylation

## Abstract

Regulated neuronal cell death plays an essential role in biological processes in normal physiology, including the development of the nervous system. However, the deregulation of neuronal apoptosis by various factors leads to neurodegenerative diseases such as ischemic stroke and Alzheimer’s disease (AD). Death-associated protein kinase 1 (DAPK1) is a calcium/calmodulin (Ca^2+^/CaM)-dependent serine/threonine (Ser/Thr) protein kinase that activates death signaling and regulates apoptotic neuronal cell death. Although DAPK1 is tightly regulated under physiological conditions, DAPK1 deregulation in the brain contributes to the development of neurological disorders. In this review, we describe the molecular mechanisms of DAPK1 regulation in neurons under various stresses. We also discuss the role of DAPK1 signaling in the phosphorylation-dependent and phosphorylation-independent regulation of its downstream targets in neuronal cell death. Moreover, we focus on the major impact of DAPK1 deregulation on the progression of neurodegenerative diseases and the development of drugs targeting DAPK1 for the treatment of diseases. Therefore, this review summarizes the DAPK1 phosphorylation signaling pathways in various neurodegenerative diseases.

## 1. Introduction

In the central nervous system, neuronal cell death is a crucial process in nerve damage and development. The death of neurons under normal physiological conditions in the adult brain is limited and adequately controlled, even in the elderly. In general, mature neurons are more resistant than immature neurons to cell death [1]. However, cell death is associated with acute and chronic neurodegenerative diseases with pathologies that include a partial loss of neurons [1,2]. Post-translational modifications (PTMs), including acetylation, methylation, ubiquitination and phosphorylation, are important for the control of cell life and death [3,4]. In particular, phosphorylation directly involved in apoptosis is a widely exploited mechanism for cellular homeostasis [5]. Moreover, phosphorylation associated with apoptosis not only leads to functional outcomes but also influences the onset of neuronal cell death related to neurodegenerative diseases [5].

Death-associated protein kinase 1 (DAPK1), as a serine/threonine (Ser/Thr) kinase, plays a critical role in the regulation of stress-induced cell death [6,7]. DAPK1 is a pro-apoptotic gene that stimulates cellular apoptosis in response to multiple internal and external apoptotic stimuli [8,9,10]. This pro-apoptotic Ser/Thr kinase is involved in caspase-dependent (i.e., apoptosis) and caspase-independent cell death processes [6,7,10]. In addition to its role in cell death, DAPK1 has been implicated in the cell cycle, tumorigenesis, tumor metastasis, inflammation, oxidative stress and neurodegeneration [7,11,12]. The PTM of DAPK1, including the phosphorylation of DAPK1, regulates its stability and activity.

This review focuses on the role of DAPK1 in neuronal cell death and neurodegenerative diseases. Furthermore, we discuss the currently understood mechanisms of neuronal cell death associated with DAPK1 phosphorylation in the damaged brain.

## 2. Death-associated Protein Kinase Family

DAPK1 is a calcium/calmodulin (Ca^2+^/CaM)-regulated Ser/Thr kinase that was originally identified by an unbiased genetic screen of an antisense cDNA expression library from HeLa cells that underwent γ-interferon (IFN-γ)-mediated cell death [13]. Since the discovery of DAPK1, four other kinases with different degrees of homology with the catalytic domain of DAPK1 have been identified [7]. Thus far, the human DAPK family is known to consist of at least five family members [14]. The two most similar family members of the DAPK family are DAPK-related protein 1 (DRP-1 or DAPK2) and zipper interacting protein kinase (ZIPK, DAPK3, or Dlk). The other two members are DAPK-related apoptosis inducing kinase 1 (DRAK1 or STK17A) and DRAK2 or STK17B, which are more distantly related [15,16,17,18,19]. In particular, DAPK2 and DAPK3 have approximately 80% homology with the kinase domain of DAPK1 [16,18]. However, DRAK1 and DRAK2 share approximately 50% identity and have rarely been studied compared to the other family members [19]. DAPK1, DAPK2 and DAPK3 are classified as a common kinase subfamily mainly due to high level of conservation within their catalytic domains located at the N-terminus [14]. However, the DAPK family members are very diverse in size and structure. DAPK1 is a large 160-kDa protein kinase that contains specific kinase domains and multiple functional domains [20]. The 42-kDa DAPK2 is composed of a Ca^2+^/CaM autoregulatory domain and a 40-amino-acid C-terminus [18]. The structure of DAPK3 is different from that of DAPK1 and DAPK2. This 55-kDa protein does not have a Ca^2+^/CaM domain but instead has a nuclear localization signal and a leucine zipper structure at the C-terminus [15]. In addition, DRAK1 (46 kDa) and DRAK2 (42 kDa) are contain catalytic domains at the N-terminus and a regulatory C-terminus for kinase activity. Both DRAKs do not have a Ca^2+^/CaM domain and are exclusively localized in the nucleus [19,21].

## 3. DAPK1 Structure

**Figure 1:**
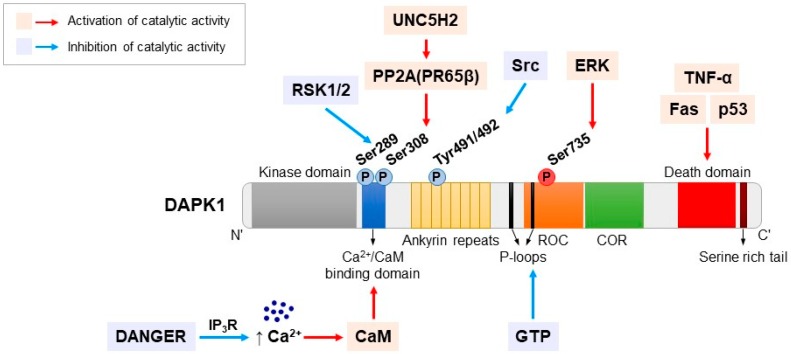
Schematic diagram of the structure of DAPK1. There are diverse molecules involved in the regulation of DAPK1 activity in neuronal cell death. The molecular mechanisms and function of DAPK1 can be regulated by multiple signals in variety of phosphorylation sites. DAPK1 activity is negatively regulated by its phosphorylation at Ser308 as well as Ser289 in the CaM autoregulatory domain and Tyr491 and Tyr492 in the ankyrin repeat domain (blue arrows). Moreover, the phosphorylation of Ser735 in the ROC-COR domain induces the catalytic activity of DAPK1 (red arrow). Furthermore, several interacting partners modify DAPK1 catalytic activity, protein-protein interactions, and pro-apoptotic activity. Phosphorylation sites in blue are negatively regulating and the one in red is activating DAPK1 activity.

DAPK1 is the largest protein kinase of in the DAPK family and is composed of 1431 amino acids [22]. It consists of multiple distinct domains and motifs, including an N-terminal kinase domain, a Ca^2+^/CaM-binding autoregulatory domain, eight ankyrin repeats, two putative P-loops, a Ras of complex (ROC) domain, a C-terminal of ROC (COR), a death domain and a Ser-rich C-terminal tail (Figure 1) [14,22]. These distinct DAPK1 domains and motifs have specific functions, such as the regulation of catalytic activity, degradation, localization and interactions with its substrates and binding partners [23].

All of the members of the DAPK family have an N-terminal kinase domain and consists of 263 residues, including 11 typical subunits with a relatively small lobe (five β-sheets and a single αC-helix) [7,24]. The catalytic domain of DAPK1 has been determined by X-ray crystallography at a resolution of 1.5 Å, which provides structural information on the activation mechanism of DAPK1, interaction with its substrates, and potential inhibitors [25,26]. DAPK1 contains an adenosine triphosphate (ATP) binding loop in its kinase domain that contains various conserved features of Ser/Thr protein kinases. The conserved lysine 42 residue (Lys42) in the ATP binding loop is required for the induction of cell death by DAPK1 kinase activity [27,28]. Furthermore, the amino terminal lobe of the kinase domain has an interaction domain for the chaperone heat shock protein 90 (HSP90). This interaction modulates the stability and activity of the kinase; therefore, inhibiting this interaction results in the degradation of DAPK1 [22]. The kinase domain of DAPK1 is followed by a 62-amino-acid Ca^2+^/CaM autoregulatory domain, which regulates DAPK1 activity by a double-locking mechanism [22]. Calmodulin (CaM) binding is required for activation and is regulated by the binding of Ca^2+^ to the autoregulatory CaM-binding region to remove this domain from the catalytic cleft [22]. In addition, the Ca^2+^/CaM autoregulatory domain regulates the activity of DAPK1 by allowing its phosphorylation at several serine sites [14]. DAPK1 contains eight ankyrin repeats of approximately 30 amino acids each. Ankyrins, which facilitate protein-protein interactions and are involved in protein degradation and/or localization, are one of the most widely existing proteins in nature [14,29,30].

Although the function of the two putative P-loops at residues 639-646 and 695-702 of DAPK1 is not well known, the second of the P-loops (residues 695-720) partially overlaps a cytoskeleton binding region, and DAPK1 binds to GTP through the second P-loop [31]. GTP binding is important for the functional activity of DAPK1. Because GTP binding negatively affects the catalytic activity of DAPK1, the deletion of the P-loop improves the cellular activity of DAPK1 [31]. The ROC-COR domain at residues 667-1228 has been identified as a cytoskeleton localization region that binds to actin microfilaments [6]. This domain promotes GTP hydrolysis to GDP through the P-loop motif in the ROC domain. GDP production by the ROC-COR domain induces conformational changes in the N-terminal domain and ultimately reduces the autophosphorylation of DAPK1 [6,31]. The ROC-COR domain plays a role in cytoskeletal localization and mediates interactions with Pin1, which is an important signaling protein that modulates diverse cellular processes, including growth-signal responses, cell-cycle progression, cellular stress responses, neuronal function and immune responses via the isomerization of proline (Pro) [32,33]. Thus, the ROC-COR domain plays an important role in the regulation of the catalytic activity of DAPK1 through protein-protein interactions. The death domain is located close to the C-terminus at residues 1321–1396 and mediates protein-protein interactions, kinase activity, and pro-apoptotic proteins such as Fas, TNF receptor type 1-associated death domain protein (TRADD), Fas-associated protein with death domain (FADD) and tumor necrosis factor (TNF) receptor [6]. The deletion of the death domain of DAPK1 significantly reduces the apoptotic ability of DAPK1 by preventing essential death domain-mediated interactions [34,35,36].

The serine-rich C-terminal tail of DAPK1 acts an autoinhibitory module that negatively regulates the putative function of the protein residues of one of the various extra-catalytic domains [35]. The death domain may be a potential target of autoinhibition by the serine-rich C-terminal tail, such as the corresponding region of the Fas/APO-1 receptor [35,36]. The overall structure of DAPK1 is unique and specific and has key functions related to cell death.

## 4. Regulation of DAPK1 by Phosphorylation

Phosphorylation plays a very important role in the regulation of DAPK1 activity (Figure 1). In particular, DAPK1 activity is negatively regulated by autophosphorylation at Ser308 in the CaM autoregulatory domain [37,38]. To activate DAPK1, its dephosphorylation at Ser308 by several phosphatases is required [39]. Protein phosphatase 2A (PP2A) is a strong candidate [40]. PP2A is an essential Ser/Thr phosphatase that regulates DAPK1 levels via stimulating the proteasomal degradation of DAPK1 [40,41]. PP2A positively regulates the activity of DAPK1 by dephosphorylating it at Ser308, and consequently, DAPK1 activation mediates many biological processes, including cell proliferation, development, and autophagic and apoptotic cell death in vivo and in vitro [40,42,43,44,45]. In addition, PP2A recruitment to the UNC5H2-dependent receptor via the structural subunit PR65β enhances DAPK1 dephosphorylation [46].

Another important DAPK1 phosphorylation site is Ser735 in the ROC-COR domain [14]. DAPK1 interacts with extracellular signal-regulated kinase (ERK); the docking sequence within its death domain is a substrate of ERK [47,48]. The role of ERK in promoting apoptosis has become increasingly clear in both in vitro and in vivo models of neuronal cell death [49]. Glutamate- or camptothecin-mediated neuronal damage requires the activation of ERK, which stimulates neuronal degeneration predominantly through plasma membrane damage [50]. The phosphorylation of Ser735 induces the catalytic activity of DAPK1 through ERK activation, which is associated with a variety of cell death mechanisms [47,51]. The phosphorylation of DAPK1 at Ser735 promotes the phosphorylation of myosin light chain (MLC), one of the substrates that induces apoptotic cell death [24,48,52]. Furthermore, the mutation of the Ser735 residue of DAPK1 to Ala (S735A) abrogates DAPK1-mediated apoptosis through binding to ERK. However, the phosphorylation-mimicking S735D mutation exhibits higher apoptosis induction than wild-type (WT) DAPK1 [47].

In addition to Ser308, another phosphorylation site, Ser289, is present in the CaM autoregulatory domain. p90 ribosomal S6 kinases (RSK1/2) are located downstream of ERK in the mitogen-activated protein kinase (MAPK) pathway [53]. ERK promotes cell death through DAPK1 phosphorylation at Ser735, whereas phosphorylation at Ser289 by RSK reduces the apoptotic activity of DAPK1 [54,55]. The relation of the effect of this phosphorylation at Ser289 to the kinase activity of DAPK1 has not been determined. However, mutations of Ser289 to phosphorylation-deficient Ala mediate enhanced apoptotic activity, whereas the phosphorylation-mimicking S289D mutation reduces apoptotic function [56,57].

Phosphorylation sites that regulate DAPK1 activity include Tyr491 and Tyr492, which are present in ankyrin repeats [56,58]. Although they are not well known for the regulation of DAPK1, it has been shown that they act as important phosphorylation sites in cancer therapy. DAPK1 is dephosphorylated at Tyr491/Tyr492 by the leukocyte common antigen-related (LAR) tyrosine phosphatase, which is involved in catalytic stimulation, apoptosis and anti-adhesion/anti-migration activity [58,59]. On the other hand, Src induces the phosphorylation of DAPK1 at Tyr491/Tyr492, which enhances intra/intermolecular interactions and the inactivation of DAPK1 [58]. This establishes a functional link between tumor progression and the DAPK1 regulation mechanism, but the link to neuronal cell death has not been determined [58,59,60].

## 5. DAPK1 and Neuronal Cell Death

Neuronal cell death occurs extensively during the development of the central nervous system as well as in pathologies associated with neuronal injury. The death of neuron results in many chronic neurodegenerative diseases due to the limited growth and replacement of adult neurons [61]. The phenotypes of neuronal cell death and its molecular mechanisms are very diverse [62]. The best known mechanism of neuronal cell death is apoptosis [63]. Other mechanisms involved in neuronal cell death include autophagy and necrosis, which are morphologically distinct from canonical apoptosis [64,65]. In many chronic neurodegenerative diseases, including AD and Parkinson’s disease (PD), there is a selective loss of specific subsets of neuronal populations over a period of years or even decades [61,66,67].

DAPK1 has an important role in various types of neuronal cell death mechanisms. DAPK1 expression in the brains of AD patients is significantly increased compared with age-matched normal subjects [68]. In addition, DAPK1 induces synucleinopathy and degeneration of dopaminergic neurons in PD [69]. The activation of DAPK1 is the cause of certain forms of apoptotic cell death, including Fas-, TNF-α-, transforming growth factor-beta (TGF-β)-, ceramide-, amyloid-beta (Aβ)-, caspase-, and p53-mediated apoptosis [10,70,71,72]. The involvement and function of DAPK1 in Fas- and TNF-α-induced apoptosis and requires the death domain [6,71,73]. Thus, the deletion of the death domain inhibits Fas- and TNF-α-induced cell death [6,71].

Furthermore, DAPK1 mediates death mechanisms through TGF-β stimulation [74]. TGF-β is a multifunctional cytokine that regulates a variety of cell functions, such as the differentiation and apoptosis of various types of cells [75,76]. TGF-β-dependent apoptosis plays a major role in the elimination of damaged or abnormal cells in normal tissues [76]. Beclin-1 is a novel BH3-only protein that interacts with the anti-apoptotic proteins of the BCL2 family, particularly Bcl-2 and its homologue Bcl-X_L_ through their BH3 domains [74]. However, TGF-β mediates pro-apoptotic events through the down-regulation of Bcl-2 and Bcl-X_L_ [76]. DAPK1 promotes the phosphorylation of Beclin-1 at Thr119 in its BH3 domain and induces the dissociation of Beclin-1 from Bcl-X_L_ [77]. Moreover, TGF-β causes cellular responses and transmits signals into cells through Smads [6]. DAPK1 activity is increased in response to TGF-β through the stimulation of Smads, particularly Smad2, Smad3 and Smad4 [76,78].

DAPK1 phosphorylates p53 at Ser23 by the direct binding of the DAPK1 death domain to the DNA binding motif of p53 (residues 270–281) [79]. In primary cortical neurons in mice, the DAPK1-mediated phosphorylation of p53 increases the transcriptional induction of pro-apoptotic genes such as Bax in the nucleus, whereas it induces necrosis through its interaction with cyclophilin D (CypD) in the mitochondrial matrix [79,80]. However, the deletion of the DAPK1 death domain or the p53 DNA binding motif that interferes with DAPK1-p53 interaction blocks neuronal death signaling mechanisms [6,79]. Therefore, the interaction between DAPK1 and p53 is a crucial signaling mechanism for the convergence of apoptotic and necrotic mechanisms [81].

DAPK1 regulates the c-Jun N-terminal kinase (JNK) signaling pathway through the binding and activation of protein kinase D (PKD) in response to oxidative stress [82]. DAPK1 activation through the PKD-JNK mechanism is characterized by caspase-independent necrotic cell death [82]. JNK1 is a stress-activated MAPK that mediates stress-induced autophagy via DAPK1 activation [83]. DAPK1-mediated JNK1 stimulates Bcl-2 phosphorylation, which drives the dissociation of Bcl-2 from Beclin-1 and the subsequent activation of autophagy [83,84,85].

DAPK1 causes cell death under pathological conditions via N-methyl-D-aspartate (NMDA) receptors in neurons [86]. NMDA receptor-mediated excitotoxicity has a key role in acute neurological disorders, such as ischemic stroke and traumatic brain injury, as well as in chronic neurodegenerative diseases, including AD [87,88]. NMDA receptors are a subtype of Ca^2+^-permeable ionotropic glutamate receptors that are known to be responsible for the neurotoxic effect of glutamate, which stimulates fast synaptic transmission in the majority of excitatory synapses in the human brain [86,89]. Native NMDA receptors are hetero-oligomeric complexes consisting of two essential GluN1 (NR1) and one or more regulatory GluN1 (NR2) subunits encoded by four genes (GluN2A-D or NR2A-D), most commonly GluN2A and GluN2B [89,90,91]. GluN2A is primarily located at synapses and preferentially mediates cell survival, whereas GluN2B is mainly located at extrasynaptic sites and is involved in cell death [92]. DAPK1 directly interacts with NMDA receptors via interacting with residues 1292–1304 in the carboxyl tail region of the GluN2B subunit [93]. Activated DAPK1 binds GluN2B, and this interaction mediates GluN2B phosphorylation at Ser1303 and enhances injurious Ca^2+^ influx via GluN1/GluN2B receptor channel conductance [93]. However, the genetic deletion of DAPK1 in vivo or in vitro protects neurons by blocking the interaction between DAPK1 and GluN2B-containing NMDA receptors [93].

DANGER, which was first identified on the basis of binding to inositol 1,4,5-trisphosphate receptors (IP_3_Rs), is a novel membrane-associated protein predicted to contain a partial MAB-21 domain [94]. DANGER physiologically binds to IP_3_Rs to improve the Ca^2+^-mediated inhibition of IP_3_R-dependent Ca^2+^ release and regulates neuronal death without affecting IP_3_ ligand binding [94]. Yeast two-hybrid assays clarifying the physiological function of DANGER, which is regulated by Ca^2+^, have identified DAPK1 as an interacting protein [95]. The direct binding of DANGER to DAPK1 inhibits the catalytic activity of DAPK1 [95,96]. A deficiency in DANGER in mouse embryonic fibroblasts and hippocampal neurons significantly elevates cell death through DAPK1 catalytic activation [95,97]. Furthermore, DANGER knockout mice exhibit considerable neurotoxicity induced by NMDA and increased brain injury after neuronal damage, such as ischemia and stroke, compared to those exhibited by WT mice [95]. Therefore, DANGER acts as a regulator of neuronal cell death through the inhibition of the DAPK1 signaling pathway by direct binding.

## 6. DAPK1 and Ischemic Stroke

Stroke is the second leading cause of death worldwide. Ischemic stroke is the most common type of stroke and occurs when there is a narrowing or blockage of arteries to the brain, leading to severe blood flow reduction [93]. Ischemic stroke is characterized by apoptotic and necrotic cell death and causes a loss of neuronal cells [98,99].

**Figure 2:**
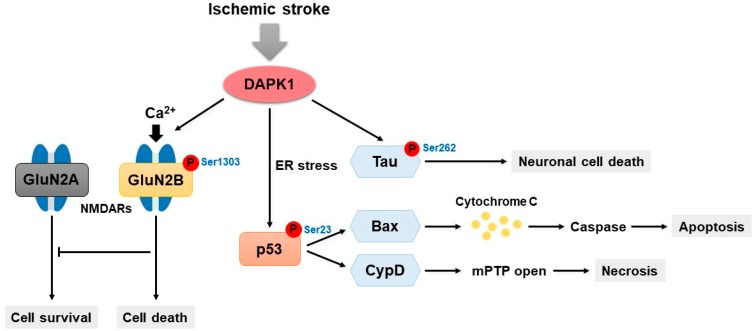
Signal transduction of DAPK1 in ischemic stroke. DAPK1 induces neuronal cell death by multiple signaling mechanisms upon ischemic stroke. Activated DAPK1 directly interacts with NMDA receptor GluN2B and phosphorylates it at Ser1303, thereby increasing neuronal cell death by enhancing Ca^2+^ influx. Upon ER stress, death domain of DAPK1 binds to the p53 DNA binding motif, followed by phosphorylation of p53 at Ser23. The interaction between DAPK1 and p53 activates both apoptotic and necrotic signaling pathways by death-related genes such as Bax and CypD through transcriptional- and mitochondrial-dependent mechanisms. Moreover, DAPK1 directly phosphorylates tau at Ser262 resulting in accumulation in the dendritic spines, which promotes neuronal cell death. mPTP, mitochondrial permeability transition pore.

DAPK1 has been shown to play a crucial role in the pathophysiology of ischemia (Figure 2). DAPK1 mRNA expression is increased following cerebral ischemia, and the function of DAPK1 is dependent on the catalytic activity of the kinase domain [100]. Moreover, DAPK1 is activated by its dephosphorylation at Ser308 following ischemia in the brain [99]. In cultured cells such as primary cortical neurons, oxygen-glucose deprivation (OGD) represents effective in vivo cerebral ischemic conditions. DAPK1 is dephosphorylated and activated following OGD, and it accelerates neuronal cell death by opening the mitochondrial permeability transition pore [101]. The activation of DAPK1 leads to endoplasmic reticulum (ER) stress and accelerates the binding of mitochondrially translocated p53 to CypD for pore opening [101]. Activated DAPK1 phosphorylates p53 at Ser23, which causes necrotic and apoptotic neuronal death [79]. In addition, increased DAPK1 activity has also been detected in an in vivo model of ischemic stroke. DAPK1 is activated through its dephosphorylation after focal cerebral ischemia in a transient middle cerebral artery occlusion (MCAO) model, which is widely used to study therapies for ischemic stroke [70]. Cell death induced by DAPK1 activation is reduced by inhibitors of calcineurin, FK506 or MK-801, or a selective NMDA receptor antagonist after OGD or MCAO [99]. DAPK1 knockout mice have a markedly reduced infarct volume of and improved neurological function after MCAO-induced cerebral ischemia [93]. Furthermore, ischemic brain injury is thought to result in a dramatic increase in the level of extracellular glutamate after the hyperactivation of NMDA receptors [102].

Activated DAPK1 directly interacts with the NMDA receptor GluN2B protein complex and phosphorylates the GluN2B subunit at Ser1303 in the cortex of ischemic stroke mice [93]. A variant of GluN2B, the GluN2B_CT_ peptide (residues 1292–1304), is membrane-permeable, specifically blocks the phosphorylation of GluN2B by DAPK1 following transient focal ischemia, and dramatically decreases the infarct size [93]. Thus, the inhibition of DAPK1 does not interfere with physiological function and prevents the excessive stimulation of NMDA receptors following stroke injury [22]. Interestingly, DAPK1 interacts with tau and directly phosphorylates tau at Ser262 in the cortical neurons of a mouse model of stroke induced by MCAO [103]. The microtubule-associated protein tau is a major concern in neurodegenerative diseases such as AD. Recently, tau has also become an important therapeutic target in acute brain ischemia [104,105,106]. DAPK1-mediated tau phosphorylation is involved in spinal cord injury and neuronal cell loss in cerebral ischemia [103]. However, the genetic deletion of the DAPK1 kinase domain in mice protects against spine damage and improves neurological functions against stroke insults [103]. Moreover, a membrane-permeable blocking peptide (TAT-R1D), which targets the DAPK1-tau binding peptide, blocks the formation of the complex and tau phosphorylation, significantly reducing the infarct area and neuronal loss induced by ischemic stroke [103]. Consequently, inhibition of tau phosphorylation by DAPK1 may be a potential therapeutic target for ischemic stroke.

## 7. DAPK1 and Alzheimer’s Disease

AD is a progressive neurodegenerative disease associated with cognitive impairment and is the most common type of dementia in the elderly, with approximately 44 million patients worldwide [107]. AD is characterized by progressive neurodegeneration and memory loss and the formation of Aβ-containing plaques and neurofibrillary tangles (NFTs) composed of hyperphosphorylated tau [27,108,109]. The Aβ peptide is a derivative of amyloid precursor protein (APP) that is generated through sequential proteolytic processing via β- and γ-secretases [110]. In addition, tau is an abnormally phosphorylated protein composed of paired-helical filaments (PHFs) and NFTs in the AD brain [111,112,113].

Interestingly, DAPK1 expression is highly up-regulated in the human AD brain [68]. DAPK1 overexpression increases tau protein stability and stimulates the phosphorylation of tau at multiple sites related to AD [68]. Similarly, DAPK1 increases the risk of PD [67,69]. The overexpression of DAPK1 in PD mice is positively correlated with neuronal synucleinopathy, dopaminergic neuron cell death and motor disabilities [69]. In contrast, genetic deletion of DAPK1 in dopaminergic neurons effectively rescues neuronal dysfunction [69]. Moreover, tau phosphorylation is pathologically associated with PD [114].

DAPK1 increases the phosphorylation of specific sites of tau, namely Thr231, Ser262, and Ser396, in neurons [68]. The phosphorylation of Thr231 is associated with tau-microtubule interactions [115]. In Thr231 tau mutants, the stability of tau is not increased by DAPK1 [68]. In addition, tau protein expression and stability is down-regulated by DAPK1 kinase-deficient mutations (K42A), whereas it is up-regulated by a constitutively active DAPK1 mutant (ΔCaM) [68]. Furthermore, DAPK1 expression is significantly enhanced by the hyperphosphorylation of hTau at Ser262 simultaneously in the cortex and CA1 and CA3 hippocampal regions of the mouse brain [112]. In contrast, the overexpression of kinase-deficient K42A and inhibits tau hyperphosphorylation, and this effect is also observed in DAPK1 knockout mice [68,112]. Moreover, DAPK1 decreases microtubule assembly and stability through the activation of microtubule-affinity regulating kinase 1 (MARK1) and MARK2, which stimulate the phosphorylation of tau at Ser262 [52,116]. In addition, the overexpression of DAPK1 remarkably increases Ser396 phosphorylation and siDAPK1 expression reduces tau phosphorylation at Ser396 in neurons [112].

DAPK1 is responsible for the phosphorylation of Pin1 [68,117,118]. Pin1 is a unique and conserved peptidyl-prolyl cis-trans isomerase (PPIase) that controls conformational changes of phosphorylated Ser/Thr-Pro motifs [33,118]. Pin1 is associated with a variety of cellular processes, such as cell-cycle progression, cellular stress responses, neuronal function, immune responses, and apoptosis [33,118]. Most notably, Pin1 dysfunction has been linked to age-dependent neurodegeneration, particularly AD [33,117]. In general, Pin1 binds to phosphorylated tau and protects against the development of tau-mediated neurodegeneration in AD by catalyzing pathogenic cis to physiologic trans conversion, particularly phosphorylated Thr231-Pro motif in tau [119]. However, Pin1 is clearly deficient in neurodegenerative disorders, including AD, but it is highly expressed in most cancers [33,118,120]. In the AD brain, Pin1 is colocalized with hyperphosphorylated tau, and its expression has an inverse relationship to tau expression [121,122]. The knockout of Pin1 induces progressive age-dependent neuropathy characterized by motor and behavioral deficits, tau hyperphosphorylation, filament formation, APP amyloidogenesis, and neurodegeneration [33,120]. DAPK1 phosphorylates the catalytic active site of Pin1, namely Ser71, thereby inhibiting cellular function and catalytic activity [32,118]. Interestingly, DAPK1 negatively regulates Pin1 by Ser71 phosphorylation and the subsequent induction of cis p-tau [118,123]. The Ser71 phospho-mimicking mutations, namely S71D and S71E, inactivate the phospho-specific PPIase activity of Pin1 and inhibit Pin1 nuclear localization and cellular function [32]. Furthermore, tau protein stability and Pin1 phosphorylation at Ser71 are significantly increased in DAPK1/Pin1-expressing cells but not in DAPK1 K42A/Pin1-expressing cells compared with vector control/Pin1-expressing cells [68]. The knockout of DAPK1 decreases the level of Ser71-phosphorylated Pin1 in mouse brains compared with WT mouse brains [68]. Thus, DAPK1-mediated Pin1 phosphorylation has a critical role that is correlated with tau stability and phosphorylation.

DAPK1 increases the phosphorylation and amyloidogenic processing of APP [27]. Interestingly, APP produces amyloidogenic fragments due to the divergence of the sequence at the internal Aβ site [124]. A fundamental abnormality that plays a pivotal role in neuronal dysfunction and death in AD modifies the proteolytic process of APP to increase the production and accumulation of neurotoxic forms of Aβ in the brain [125]. The excessive accumulation of Aβ, the major component of amyloid plaques, is a crucial step in the pathogenesis of AD [110]. DAPK1 not only promotes tau protein accumulation and phosphorylation but also participates in the process of amyloidogenic APP production [72,112]. DAPK1 overexpression significantly increases human Aβ_40_ secretion; however, DAPK1 K42A mutations do not affect Aβ_40_ secretion compared with that of WT DAPK1 in a neuronal culture model [27]. Moreover, DAPK1 increases the secretion of Aβ_42_ in cells stably overexpressing the Swedish mutant form of APP (APPswe), thus shifting APP processing toward the β-secretase-mediated pathway [27]. However, the inhibition of DAPK1 expression or catalytic activity by knockdown significantly reduces Aβ secretion [27]. DAPK1 interacts with APP and triggers APP phosphorylation at the Thr668 site [27]. The phosphorylation of Thr668 is increased in the AD brain [126]. Furthermore, DAPK1 regulates APP phosphorylation through JNK3 and GSK-3β activation [27]. The knockout of DAPK1 attenuates APP-mediated amyloidogenic processes and decreases Aβ generation [27]. In contrast, DAPK1 shifts APP processing toward the non-amyloidogenic pathway and decreased Aβ production in Tg2576 APPswe-overexpressing mice [27].

**Figure 3:**
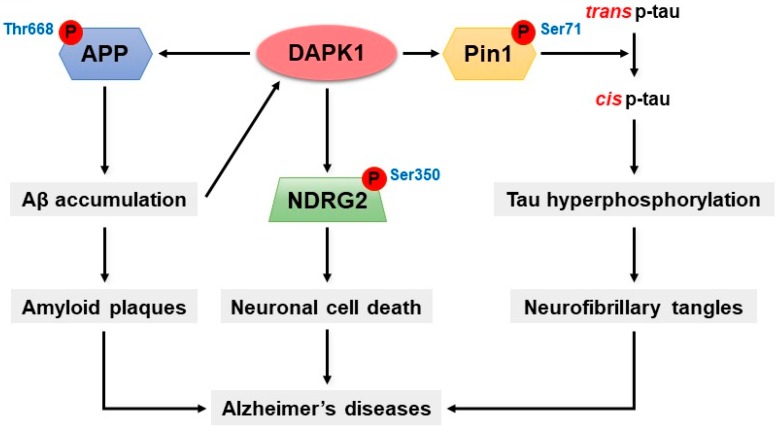
The molecular mechanism of DAPK1 in AD. The activation of DAPK1 triggers the phosphorylation of APP at Thr668, shifting APP processing toward the amyloidogenic pathway and Aβ production. Moreover, DAPK1 considerably inactivates Pin1 activity through its phosphorylation at Ser71. The inactivation of Pin1 increases cis p-tau and induces the hyperphosphorylation of tau. Furthermore, DAPK1 regulates NDRG2 phosphorylation at Ser350, which may lead to AD through significant cell death.

Through phospho-peptide library screening, N-myc downstream-regulated gene 2 (NDRG2) has been identified as a novel substrate for DAPK1 [72]. NDRG2 is involved in various biological activities, including cell proliferation, differentiation, and apoptosis [127]. Both the mRNA and protein levels of NDRG2 are significantly increased in AD-affected brains, and NDRG2 is related to the mechanism of onset in the human brain [127,128,129]. NDRG2 plays an important role in the regulation of neuronal cell death and AD through a direct interaction with DAPK1 [72]. The C-terminal domain of NDRG2 specifically binds to DAPK1 through its ROC-COR domain [72]. DAPK1-mediated NDRG2 phosphorylation activates cell death in vivo and in vitro via a caspase-dependent mechanism [72]. DAPK1 directly phosphorylates NDRG2 at Ser350 and promotes neuronal cell death through the cleavage of caspase-3 [72]. Furthermore, the levels of DAPK1 and Ser350-phosphorylated NDRG2 are also significantly increased in human AD brains [72]. While a decrease in phosphorylation of NDRG2 at Ser350 has been detected in DAPK1 knockout mice and DAPK1 K42A-expressing cells, DAPK1 ΔCaM-expressing cells exhibit significantly increased NDRG2 phosphorylation compared with that of WT controls [72]. Moreover, ceramide- or Aβ-induced DAPK1 overexpression increases neuronal cell death through NDRG2 phosphorylation in a caspase-dependent manner [72]. The deletion of DAPK1 by Tg2576 APPswe-overexpressing mice inhibits ceramide-induced NDRG2 phosphorylation and decreases neuronal death in the brain [72]. In summary, DAPK1 may be a key regulator of the pathogenesis of AD by regulating the phosphorylation of Pin1, tau, APP, and NDRG2 (Figure 3).

## 8. DAPK1 as A Potential Target for Neurodegenerative Diseases

DAPK1 is a potential molecular target for neuronal cell death, and certain inhibitors of DAPK1 have indicated the potential of new therapeutic strategies for treating neurodegenerative diseases such as ischemic stroke and AD (Table 1).

A specific small-molecule inhibitor of DAPK1 (IC_50_ = 13 μM) is an alkylated 3-amino-6-phenylpyridazine that is known to significantly attenuate brain damage after ischemic stroke [130,131]. Initially, this small-molecule DAPK1 inhibitor was synthesized to validate potential drug discovery targets for acute brain injury and was tested in vivo [130]. A single administration of the molecule was shown to reduce brain damage in tissues following both acute and sustained injury in animals [130]. Accordingly, this molecule may offer a new treatment for early programmed cell death in acute brain injury [130]. Another DAPK1 inhibitor is compound 6 (C6), which has the structure 4-(pyridin-3-ylmethylene)oxazol-5(4H)-one [132]. This potent and selective inhibitor of DAPK1 activity (IC_50_ = 69 nM) was identified via a structure-based virtual screening approach [132]. The binding mode of the C6 to DAPK1 was predicted using an in-house docking calculation program (CONSENSUS-DOCK), and the structure–activity relationship was analyzed using solvated interaction energy calculations at the ATP binding site [132]. The high selectivity of C6 for DAPK1 allows it to potentially contribute to the treatment of ischemic stroke [132]. Treatment with C6 considerably attenuates Aβ-induced cell death through the inhibition of Aβ_40_ and Aβ_42_ secretion via the down-regulation of APP phosphorylation [27,72].

Peptide-based methods of DAPK1 inhibition rapidly and reversibly knockdown endogenous proteins in situ [133]. The DAPK1 binding domain of GluN2B and the chaperone-mediated autophagy-targeting motif (CTM) are target peptides for lysosomal degradation [133]. The knockdown of DAPK1 by peptide-base methods can protect primary neurons and neurons in ischemic brains induced by MCAO against NMDA receptor-independent oxidative stress [133]. This unique endosome-lysosome system can easily degrade cellular proteins by reducing the level of protein and promoting the development of effective therapeutics [133]. Another DAPK1 inhibitor is pyrazolo[3,4-d]pyrimidinone (HS38), which inhibits DAPK1 activity at nanomolar concentrations (IC_50_ = 200 nM) in an ATP-competitive manner [134]. It was identified through a fluorescence-linked enzyme chemoproteomic strategy (FLECS) developed to rapidly identify inhibitors of any purine-using protein [134]. Although its function is not known in neurons, HS38 can provide important information for the development of drugs for neuronal disorders. In addition, an inhibitor of DAPK1 was found using the 1-anilinonaphthalene-8-sulfonic acid (ANS) competitive binding assay [135]. Morin is a flavonoid that has a high affinity for DAPK1 due to the interaction between 2′-OH and the K42 residue of DAPK1 [135]. The IC_50_ value of morin against DAPK1 is 11 μM, and morin moderately inhibits the catalytic activity of DAPK1. Although its role in neurons has not been elucidated, morin is a strong candidate for drug development [135].

In addition, a novel small-molecule imidazo-pyramidazine inhibitor was identified by a GluN2B peptide-based method using a caliper microfluidics capillary electrophoresis system, and this molecule was found to have a potent inhibitory effect on DAPK1 (IC_50_ = 0.247 μM) [136]. Recently, the potential lead compound 11 has been developed that interrupts DAPK1-GluN2B interaction [137]. This inhibitor has a promising inhibitory effect on DAPK1 (IC_50_ = 0.03 μM) and is highly selective for the ATP binding sites and substrate recognition motifs, including Gly-Glu-Leu (GEL) and Pro-Glu-Asn (PEN) [137]. More recently, dual inhibitors targeting DAPK1 as well as macrophage colony-stimulating factor 1 receptor (CSF1R) have been developed as potential agents to inhibit tau aggregates and neuroinflammation [138]. CSF1R plays a role in regulating the survival and proliferation of microglial cells, and its inhibition leads to a reduction of neuroinflammation and neuronal damage [138]. The dual inhibitor compound 31 (3,5-dimethoxy-*N*-(4-(4-methoxyphenoxy)-2-((6-morpholinopyridin-3-yl)amino)pyrimidin-5-yl)benzamide), is a unique and selective inhibitor of both DAPK1 (IC_50_ = 1.25 μM) and CSF1R (IC_50_ = 0.15 μM) [138]. More importantly, it has high blood-brain barrier (BBB) permeability in the absence of toxicity and significantly decreases tau aggregation (IC_50_ = 5.0 μM) in vitro [138].

Interestingly, there is a conformational-specific tau antibody that influences DAPK1 activity. As mentioned above, DAPK1 specifically inhibits Pin1 activity and subsequently induces of cis p-tau, particularly at Thr231 [33,118,123]. A rabbit polyclonal antibody and a mouse monoclonal antibody (clone 113) that can distinguish cis from trans tau have been generated [139,140]. Cis p-tau is detected in both human patients and animal models of a variety of neurodegenerative diseases such as AD, chronic traumatic encephalopathy and traumatic brain injury [141]. Thus, this cis p-tau antibody can be used to block tau pathology and prevent neural degeneration [142]. Overall, the development of various inhibitors of DAPK1 activity in neurodegenerative diseases has contributed greatly to the discovery of therapeutic drugs for these diseases.

## 9. Conclusions and Perspective

In a variety of neurodegenerative diseases, DAPK1 is the major key protein kinase that regulates cell death in the brain. DAPK1 is a well-known protein that plays a crucial role in neuronal cell death, including apoptosis and autophagy, through the death signaling pathway. The most important mechanism that regulates DAPK1 catalytic activity is phosphorylation. DAPK1 consists of multiple domains with special phosphorylation sites, such as Ser308, that are important for catalytic activation related to pathology in neurons. Therefore, DAPK1 inhibitors are likely to be very potent therapies because DAPK1 activity is markedly elevated in ischemic stroke and AD brains. Most of the DAPK1 inhibitors that have been developed have been shown to improve neurological function. However, there are still many open questions about the development of DAPK1 inhibitors as therapies. Further research is needed to clinically validate these DAPK1 inhibitors. To date, there is no potent treatment for neurological disorders in humans, but the treatment of neurodegenerative diseases with DAPK1 inhibitors should be strongly considered. Thus, animal experiments with DAPK1 inhibitors should be given priority. Since in vivo studies of most identified inhibitors have been limited, it is necessary to demonstrate the efficacy of these inhibitors in animals. Furthermore, the clinical utility of these inhibitors should be tested. The development of DAPK1 inhibitors is still at an early stage, and the development of new drugs with therapeutic effects on humans may be challenging. In addition, it is important to determine the underlying mechanism and function of DAPK1 to provide knowledge for novel drug therapies. DAPK1 is a key regulator that binds to numerous substrates involved in cell death. A better understanding of the mechanism of DAPK1 and its regulation under different pathological conditions, should help to define the main substrates for therapy. A further approach is to demonstrate the function of various PTMs of DAPK1 other than phosphorylation. The function of DAPK1 occurs mainly through phosphorylation, but PTMs such as acetylation and methylation may also play a role. Therefore, subsequent studies are also needed to clarify how signals are transmitted and how DAPK1 interacts with its binding partners. This study provides an opportunity for novel drug development based on the role of DAPK1, which is not yet fully understood.

## Figures and Tables

**Table 1:** Potential inhibitors related with DAPK1.

Molecule	IC_50_	Function of Inhibitor in Neuron	Ref.
Alkylated 3-amino-6-phenylpyridazine	13 μM	Reduction of brain injury and treatment of neuronal cell death	[130,131]
4-(pyridin-3-ylmethylene)oxazol-5(4H)-one	69 nM	Potential treatment of ischemic stroke and attenuation of Aβ-induced cell death	[27,72,132]
Peptide-based DAPK1 inhibitor targeting GluN2B and CTM	N/A	Protection of neuron in ischemic brain	[133]
Pyrazolo[3,4-d]pyrimidinone (HS38)	200 nM	N/A	[134]
Morin	11 μM	N/A	[135]
Imidazo-pyramidazine	247 nM	N/A	[136]
Peptide-based DAPK1 inhibitor targeting GEL and PEN	30 nM	N/A	[137]
3,5-dimethoxy-*N*-(4-(4-methoxyphenoxy)-2-((6-morpholinopyridin-3-yl)amino)pyrimidin-5-yl)benzamide	1.25 μM	Reduction of tau aggregation	[138]
Conformational-specific *cis p*-tau antibody	N/A	Reduce tau pathology and prevent neural degeneration	[33,118,123,139,140,141,142]
